# Resistin levels in the gingival crevicular fluid among diabetic and non-diabetic chronic periodontitis patients

**DOI:** 10.6026/97320630017899

**Published:** 2021-10-31

**Authors:** Keerthidaa Govindaraj, Uma Sudhakar, S Bhuminathan, Jayamathi Govindaraj

**Affiliations:** 1 MGM Health care, Nelson Manickam Road, Chennai, India; 2 Thai Moogambigai Dental College and Hospital, Maduravoyal, Chennai, India; 3 Biochemistry Department, Shree Balaji Dental College & Hospitals, BIHER, Pallikaranai, Chennai, India

**Keywords:** Resistin, chronic Periodontitis, diabetes mellitus, gingival crevicular fluid

## Abstract

It is of interest to document theresistin levels in chronic periodontitis patients (CP) with or without type 2 diabetes mellitus (T2DM) in the gingival crevicular fluid (GCF).The expression of resistin was significantly higher in chronic periodontitis when compared to the periodontally healthy groups. Resistin levels were high in CP and T2DM. Therefore, GCF resistin levels is of interest as a potential incendiary marker for periodontitis with T2DM.

## Background:

Periodontitis, an interminable provocative issue is one of the most pervasive human microbial infections influencing 10-15% of total population. The essential etiology is because of the communication between the pathogenic microorganism and host protective mechanisms [1,2]. Resistin was first portrayed in 2001 during a quest for genes that initiated adipocyte differentiation and it was down directed in full grown adipocytes during introduction to thiazolidinediones [1]. Resistin a cysteine-rich molecule, otherwise called as FIZZ3 (found in inflammatory zone-3) or ADSF (adipocyte specific secretory factor), belongs to the group of adipokines whose protein was found initially in mouse adipose tissue [3-4]. It acts as a pro-inflammatory factor and upgrades the discharge of pro-inflammatory cytokines, TNF- alpha and IL-12, and induced the nuclear translocation of NF-kappa B transcription factor [5]. It potentiated TNF-alpha, IL-6 and monocyte chemo attractant protein-1 (MCP-1) production [6]. Studies have shown resistin has been concentrated in the gingival crevicular liquid (GCF) as an inflammatory mediator in experimental gingivitis in humans. Resistin may hold an incentive as an inflammatory mediator since it has been related with insulin resistance and periodontitis [7]. Therefore, it is of interest to document the resistin levels in chronic periodontitis patients (CP) with or without type 2 diabetes mellitus (T2DM) in the gingival crevicular fluid (GCF).

## Materials & Methods:

The investigation was directed from the outpatient clinics of the Department of Periodontics, Thai Moogambigai dental college, Chennai, India. The examination included 60 individuals and the patients were divided into four groups (20 each) as group I (healthy), group II (generalized CP), group III (generalized CP without T2DM) and group IV (generalized CP with T2DM).

The inclusion criteria for the healthy periodontium were subjects having acceptable oral hygiene, no bleeding on probing, no visual indications of gingival inflammation, the plaque Index Score of "0", the gingival Index score of "0", probing pocket depth and clinical attachment level of ≤3 mm [8]. The inclusion criteria for generalized chronic periodontitis were subjects showing Presence of inflammatory changes in periodontal tissues, PI score ≥1,GI ≥1,PPD ≥ 5mm (30 %of the sites), CAL ≥ 4mm (30 %of the sites), Positive for BOP and Radio graphic evidence of bone loss. The inclusion criteria for type 2 diabetes mellitus were previously diagnosed with T2DM as obtained from their medical history, duration of diabetes for more than 6 months, RBS levels ≥ 200 mg/dl, good or fair glycemic control (confirmed with HbA1C values ≥8%) Patients having aggressive forms of periodontal disease, history of periodontal treatment received in the past six months, underlying systemic diseases, patients on high dose steroid therapy, pregnancy, lactation and smokers were excluded from this study.

The clinical periodontal parameters including, plaque index (PI), gingival index (GI), probing pocket depth (PPD) and clinical attachment level (CAL) were assessed for the subjects. Midbuccal, distobuccal, mesiobuccal and palatal sites in each tooth were recorded for PI [9]. The buccal, mesial, distal and lingual gingival areas were inspected for GI [10]. PPD and CAL were estimated in millimetres and were assessed in all the teeth at six sites and CAL was determined from cemento-enamel junction to periodontal pocket base [11,12].

The investigation convention was done as per moral principles and was endorsed by the Institutional Review Board ((Dr. MGR DU/TMDCH/RES/2016/2582),). The purpose and type of the evaluation was explained to the subjects verbally and educated assent was acquired from the patients. Radiographic bone loss was recorded for each patient by intra oral periapical radiographs utilizing long cone method or utilizing ortho pantomogram (OPG) to differentiate chronic periodontitis patients from healthy group.

## GCF resistin analysis:

Supra gingival scaling was done a day before GCF collection. A volume of 1µl (microlitre) was procured from each test site by an extracrevicular approach, utilizing volumetric microcapillary pipettes. The collected GCF was immediately transferred to Eppendorf tubes . The GCF was stored at - 20°C until the hour of assay. The resistin levels were evaluated using Enzyme- Linked Immunosorbent Assay (ELISA), which comprised of 96 well plates coated with human resistin specific antibody. Standards and sample solution were added to the wells,resistin present in the sample binds to the antibody. Biotinylated anti-human resistin antibody was poured and by washing unbound biotinylated antibody was evacuated. At that time, horseradish peroxide conjugated streptavidin was poured, trailed by tetramethyl benzidine solution into the wells. Blue colour was observed, which after addition of stop solution changed to yellow. The colour intensity was correlated with the resistin levels and it was pursued in ELISA reader. The resistin levels were expressed in ng/ml.

## Statistical analyses:

The outcomes acquired were recorded for the case sheets and later entered in MICROSOFT EXCEL in independent sheets for each groups. To analyze the data SPSS (IBM SPSS Statistics for Windows, Version 23.0, Armonk, NY: IBM Corp. Released 2015) was utilized. 5% (α = 0.05) was fixed as Significance level. To check for the normal distribution of the data, the normality tests, Kolmogorov – Smirnov and Shapiro wilks tests were performed. Along these lines both parametric and non parametric tests were applied for examination. For inter group comparison, One way ANOVA was applied.

## Results and Discussion:

The mean and standard deviation (S.D) of the clinical parameters such as PI, PPD,CAL and biochemical parameters such as RBS, HbA1C, Resistin are depicted in Table 1.On comparison of clinical parameters the difference was found to be statistically significant (p<0.0001). In the present study, the mean plaque index values were high in group IV (2.76 ± 0.241) followed by group II (1.993 ± 0.263) & Group III (0.526 ± 0.234) and compare to Group I (0.313 ± 0.273). Very strong Positive correlation and a statistically significant difference was observed between resistin with RBS, HbA1C and clinical parameters and it was also statistically significant (p<0.0001*).

Oral cavity and systemic diseases are two entities that can influence one another [9]. Periodontitis is considered to be the sixth complication of diabetes, and a bidirectional relationship has been built up between diabetes and periodontitis, wherein one can impact the other [8].Recent evidences stated that resistin participates in inflammatory process which inturn displays proinflammatory cytokines, suggesting a role for this cytokine in linking periodontal disease and diabetes.[13]. In the present study, the mean of clinical parameters were high in group IV followed by group II & Group III and compared to Group I and was statistically significant (p <0.0001).

Gingival index was selected as a method for evaluating the severity and quantity of gingival inflammation indicated the presence of active periodontal disease. The mean GI was higher in Group IV followed by group II and group III than in Group I and was statistically significant (p <0.001). This was in accordance as shown elsewhere [8 & 12] where they showed that the mean PI and GI scores were high in diseased subjects than in the healthy. This was attributable to the fact that, plaque being the primary and essential disease initiating factor that results in a transition from health to gingivitis and gingivitis when left untreated could progress to periodontitis [14].

The level of glycemic control is of key importance in determining increased risk. The glycemic parameters RBS and HbA1C values were higher in group IV (T2DM with CP) followed by Group III (T2DM). The mean values of RBS is significantly high in Group IV and III than in Group II and Group I. HbA1c is considered as a beneficial indicator of long –term homeostasis, reflecting an average blood glucose concentration for the past 2-3 months [15]. In the present study, mean HbA1C values were higher in Group IV & Group III than in Group II and Group I. Both RBS and HbA1C values were found to be statistically significant (p < 0.001). This was in concurrence to a study by Gokhale et al. [8]. The elevated inflammatory state and hyperglycemia induces the activation of pathways that increases the inflammation, oxidative stress and apoptosis to accelerate the dysfunction in periodontal tissues which is already present because of insulin resistance. This increases the susceptibility to infection that could clearly accelerate the destruction of the periodontal tissues [16]. A very strong positive correlation and a statistically significant difference were observed between HbA1C and RBS levels. But there was only a mild positive correlation between HbA1C and clinical parameters which reflected the inflammatory state of the periodontium among the four groups. Demmer RT et al(2008) [17] who reported that there was a statistically significant difference observed between HbA1C with CAL. Another study done by Katia et al(2017) [18], who reported that the HbA1C levels were greater in individuals with greater PPD at base line. They concluded that a possible association does exist between the levels of HbA1c and the risk of development of periodontal pathologies.

The regression of resistin with regard to individual groups showed that the inflammatory parameters had a predictive potential. It is therefore stated with caution that resistin levels in GCF showed an overall positive correlation to PI, GI, PPD, RBS, and HbA1c. This finding was in agreement with an earlier study done by Mealey BL et al (2007)& Tsai C et al(2002)[19-20]. The currently available literature proposes that the degree of resistin are increased in the subjects with chronic periodontitis compared to the clinically healthy controls. In this way, periodontitis might prompt to development of type II diabetes or diabetes might impact the occurrence or progression of periodontitis. However, there are very few studies in the field of Periodontics, tending to the relation between chronic periodontitis and resistin, which goes about as a biomarker, "the connecting link between periodontitis, obesity, and diabetes". This investigation gives a definitive association of GCF resistin in patients with Chronic Periodontitis and Type 2 Diabetes Mellitus.

## Conclusion:

Data is significant in understanding their role in the changing dynamics of periodontal disease progression. Resistin, is a potential marker for periodontitis with Diabetes. However, caveats regarding its association with metabolic parameters need to be assessed. Therefore further data can help in periodontal therapy and glycemic control on the levels of resistin in GCF. Moreover, creating therapeutic strategies can have wide spread applications given the multitude of inflammatory conditions in which resistin is shown to take part, including periodontitis.
